# Inflammatory Bowel Disease in Children of Middle Eastern Descent

**DOI:** 10.1155/2014/906128

**Published:** 2014-06-02

**Authors:** Christina Mai Ying Naidoo, Steven T. Leach, Andrew S. Day, Daniel A. Lemberg

**Affiliations:** ^1^School of Women's and Children's Health, University of New South Wales, Sydney, NSW 2052, Australia; ^2^Department of Gastroenterology, Sydney Children's Hospital, High Street, Randwick, Sydney, NSW 2031, Australia; ^3^Department of Pediatrics, University of Otago (Christchurch), Christchurch 8140, New Zealand

## Abstract

Increasing rates of inflammatory bowel disease (IBD) are now seen in populations where it was once uncommon. The pattern of IBD in children of Middle Eastern descent in Australia has never been reported. This study aimed to investigate the burden of IBD in children of Middle Eastern descent at the Sydney Children's Hospital, Randwick (SCHR). The SCHR IBD database was used to identify patients of self-reported Middle Eastern ethnicity diagnosed between 1987 and 2011. Demographic, diagnosis, and management data was collected for all Middle Eastern children and an age and gender matched non-Middle Eastern IBD control group. Twenty-four patients of Middle Eastern descent were identified. Middle Eastern Crohn's disease patients had higher disease activity at diagnosis, higher use of thiopurines, and less restricted colonic disease than controls. Although there were limitations with this dataset, we estimated a higher prevalence of IBD in Middle Eastern children and they had a different disease phenotype and behavior compared to the control group, with less disease restricted to the colon and likely a more active disease course.

## 1. Introduction

Inflammatory bowel disease (IBD) is a chronic, relapsing, idiopathic inflammation of the gastrointestinal tract. Subtypes of IBD include Crohn's disease (CD), ulcerative colitis (UC), and inflammatory bowel disease unclassified (IBD-U). The majority of epidemiological studies on the incidence and prevalence of IBD relate to the adult population [1]. Historically, Europe and North America have been considered high incidence areas while Asia, Africa, and the Middle East have been considered low incidence areas [2, 3]. Emerging data has suggested that the incidence of IBD is increasing globally in both developed and developing countries [3]. One study from Central Saudi Arabia on the epidemiology of juvenile onset IBD estimated an incidence of 0.5 per 100 000 per year and a prevalence of 5/100 000 [4]. While this is significantly lower than the incidence rates of 11.43/100 000 per year reported in North America [5], comparison with older data nevertheless suggests an increasing incidence [4].

The emergence of chronic inflammatory diseases such as IBD has been closely linked to social and economic development [6] and it has been postulated that the “Westernisation” of society accounts for the increasing incidence of IBD in countries where it was once considered rare [3]. The importance of ethnic, racial, and geographic factors in IBD is illustrated by the considerable literature citing varying risks of developing IBD in different ethnic populations. It has been well established that the Ashkenazi Jewish population have a higher risk of developing IBD than other ethnic groups [7, 8]. Several studies also show that ethnic groups with low rates of IBD in their home country have a much higher incidence of IBD following immigration to Western countries [9]. For instance, studies on migrant populations found a higher incidence of IBD in South Asians than Non-South Asians in the pediatric population of British Columbia and the adult population of Leicester [9–11].

Recent studies have found the overall incidence rate of IBD in Australia to be among the highest reported [12]. In addition, Phavichitr et al. [13] found that the incidence of pediatric CD in Victorian children rose from 0.128 per 100 000 per year in 1971 to 2.0 per 100 000 per year in 2001. However, there are no reports on the epidemiology of IBD in specific ethnic groups in Australia. Given the fact that Australia is a multicultural society with significant emigration of families from the Middle East this may be relevant. Therefore the objectives of the current study were to examine the clinical characteristics and management of IBD in children of Middle Eastern descent diagnosed at the Sydney Children's Hospital Randwick (SCHR).

## 2. Methods

### 2.1. Patients and Data Collection

A retrospective chart review was undertaken on all patients identified as being of Middle Eastern ethnicity on the SCHR IBD database. IBD specific data collection began at SCHR in 1987 and data was complete up to the year 2011 at the time of review. At diagnosis, parents were requested to provide a familial history including familial ethnicity. Children were considered to be of Middle Eastern ethnicity if one or both parents self-identified as being of Middle Eastern ethnicity or from any of the following countries: Egypt, Iran, Iraq, Israel, Jordan, Kuwait, Lebanon, Oman, Qatar, Saudi Arabia, Syria, United Arab Emirates, West Bank and Gaza, and Yemen. Children with Israeli ancestry and Jewish ethnicity were not included in this study due to the well-defined predisposition to IBD in this population. A control group of patients of non-Middle Eastern descent was also identified from the SCHR IBD database: these were matched to patients of Middle Eastern descent according to age at diagnosis, gender, and disease type. The project was approved by the South Eastern Sydney and Illawarra Area Health Service Research Ethics Committee.

Information collected at diagnosis for the study and control groups included family history of IBD in first degree relatives, smoking exposure history, residential postal code, age at diagnosis, symptoms at presentation, duration of symptoms at presentation, specific blood tests (erythrocyte sedimentation rate (ESR), C-reactive protein (CRP), platelets, albumin, haematocrit, alanine transaminase (ALT), aspartate transaminase (AST)), disease location, extraintestinal manifestations, pediatric Crohn's disease activity index (PCDAI) or pediatric ulcerative colitis activity index (PUCAI) score, height, and weight. Disease management information was also collected including whether they received the following treatments: exclusive enteral nutrition (EEN), corticosteroids, aminosalicylates, thiopurines, methotrexate, biological, tacrolimus, or surgical intervention.

Disease location was classified according to the Montreal classification of L1 (terminal ileum), L2 (colon), L3 (ileocolonic), and L4 (upper gastrointestinal (GI)) for CD [14]. Symptoms at presentation were grouped under the following categories: abdominal pain, diarrhoea, mucus and/or blood in stools, weight loss, per rectal bleeding, and loss of appetite. Height and weight measurements at diagnosis were converted to height for age *z*-scores and weight for age *z*-scores using the Centre for Disease Control application EpiInfo, based on the CDC-2000 charts.

### 2.2. Estimation of Point Incidence and Point Prevalence

Point incidence and point prevalence were calculated for both the Middle Eastern study group and the control group for the SCHR catchment area. For this purpose the catchment of the SCHR was defined as the Local Government Areas (LGA) of the South Eastern Sydney and Illawarra Area Health Service, which includes Botany, Hurstville, Kogarah, Randwick, Rockdale, Sutherland, Sydney, Waverley, Wollongong, and Woollahra. Population information for the LGAs was obtained from the Australian Bureau of Statistics 2006 census data. Ancestry information in the 2006 census data was collected by similar means to data in the SCHR database, where ancestry was defined by self-reporting of familial ancestry and birthplace. The census data was sorted by ancestry, LGA, and age (0–16 years). The SCHR IBD database was used to identify patients who resided in the defined catchment area and had active inflammatory bowel disease in 2006 to calculate point prevalence. The database was also used to identify those patients within the catchment area who were diagnosed in 2006 to calculate the point incidence.

The remoteness area category was calculated for each patient from residential postal codes based on the Australian Standard Geographical Classification-Remoteness Area (ASGC-RA). The ASGC-RA is a hierarchical classification system of geographical areas developed by the Australian Bureau of Statistics (ABS) that provides a common framework of statistical geography. The categories used were RA1 (major cities of Australia), RA2 (inner regional Australia), RA3 (outer regional Australia), RA4 (remote Australia), and RA5 (very remote Australia).

### 2.3. Statistics

Statistical analysis was carried out using Graph Pad Prism 5. A Fisher's exact test was used to compare the two groups with regard to smoking exposure history, family history, symptoms at presentation, extraintestinal manifestations, and disease location. The management of IBD was analysed by Fisher's exact tests, with UC and CD being analysed separately. A chi-squared test was used to compare ASGC-RA scores. An unpaired *t*-test was used to compare platelets, albumin, haematocrit, PCDAI, height for age *z*-scores, and weight for age *z*-scores between the two groups. The Mann-Whitney test was used to compare the groups for ESR, CRP, ALT, and AST. Results were considered significant if *P* < 0.05. The relative risk (RR) was calculated for incidence and prevalence; a result was considered significant if the confidence intervals (CI) did not embrace a relative risk of one.

## 3. Results

### 3.1. Study and Control Populations, Demographics, and Disease Characteristics

Of the 441 patients on the SCHR IBD database, 35 (7.9%) were identified as being of Middle Eastern ethnicity. However, 11 of the 35 were excluded from this retrospective study as files for these patients were unavailable. Therefore a final cohort of 24 patients of Middle Eastern ethnicity (both parents of Middle Eastern ethnicity) and 24 non-Middle Eastern controls were included in this study.

Of the 24 patients of Middle Eastern ethnicity, 14 (58.3%) had CD, 7 (29.2%) had UC, and the remainder had IBD-U (Table 1). Fifteen (62.5%) of the group were male: 9 of these had CD and 4 had UC and 4 had IBD-U. Twenty (83.3%) patients were born in Australia, 2 (8.3%) were born in Lebanon, and 2 were born in the USA. The mean age at diagnosis overall was 9.8 years (range, 0.7–15.7). Four patients (16.6%) were diagnosed under the age of five years and 11 patients (45.8%) were diagnosed before the age of 10 years. There was mean of 92 days (range 2–3227 days) between date of diagnosis of the Middle Eastern ethnicity patients and their matched controls. All but one of the control patients were born in Australia. Data on consanguinity was unavailable.

In those children identified as Middle Eastern ethnicity, 16 had parents identified as Lebanese, 3 Egyptian, 2 Turkish, and 1 Algerian, and 2 parents did not provide a country of birth but self-identified as Middle Eastern ethnicity. Of the controls, 18 had parents identified as Caucasian, 2 Indian, and 1 Caucasian-Jewish, and 3 did not provide a country of birth but self-identified as non-Middle Eastern ethnicity. There was no difference in family history of IBD in first-degree relatives of Middle Eastern (5/22; 2 unknown; 22.7%) and control (2/22; 2 unknown; 9.1%) patients. There was no difference in smoking exposure history between the two groups, Middle Eastern (7/21; 33.3%) and control patients (5/19; 26.3%). All Middle Eastern patients (24/24; 100%) were living in RA1 (major city), while the controls had fewer patients (17/24; 70.8%) in RA1 and a greater distribution over RA2 (4/24; 16.7% inner regional Australia) and RA3 (3/24; 12.5% outer regional Australia) areas (*P* = 0.017).

Symptom duration prior to diagnosis did not vary between the Middle Eastern (median 8, range 1–208 weeks) and control groups (median 16, range 2–104 weeks) (*P* = 0.37). Abdominal pain and diarrhoea were the most common symptoms at presentation for both groups (Table 2). Erythrocyte sedimentation rate (ESR) at diagnosis was more elevated in Middle Eastern children compared to controls (*P* = 0.02); however, all other standard blood results were similar in both groups (Table 3). ALT and AST values were lower in the Middle Eastern group compared to the control group (*P* = 0.03 and *P* = 0.02, resp.) (Table 3).

**Figure 1:**
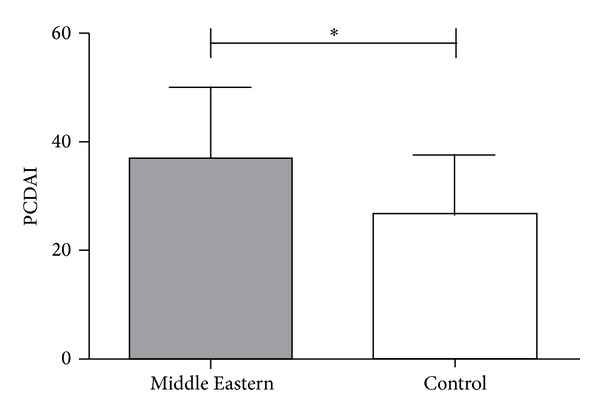
The pediatric Crohn's disease activity index (PCDAI) at diagnosis was significantly higher (*P = 0.033) in children identified with Middle Eastern descent compared to children of non-Middle Eastern decent.

PCDAI scores at diagnosis were significantly higher in the Middle Eastern group (mean 37, SD 13) compared to the control group (mean 27, SD 11; *P* = 0.033) (Figure 1). There was insufficient data to analyse PUCAI scores. Height for age *z*-score and weight for age *z*-scores at diagnosis were similar between the groups. Two (8.3%) of the Middle Eastern patients and one (4.2%) control had height for age *z*-score indicating stunted growth (< −2 SD). There was a lower incidence of colonic disease (L2) (*P* = 0.01) in the Middle Eastern group with CD compared with the control group (Table 4). Upper GI disease was present in 10 (71%) of the controls and 13 (93%) of the Middle Eastern CD patients. Terminal ileal location (L1), ileocolonic disease (L3), and upper GI tract involvement (L4) were similar in both patient groups (Table 4). There was no difference between the groups for disease location in UC as most patients in both groups had pancolitis (E3).

### 3.2. Estimated Point Incidence and Point Prevalence

**Figure 2:**
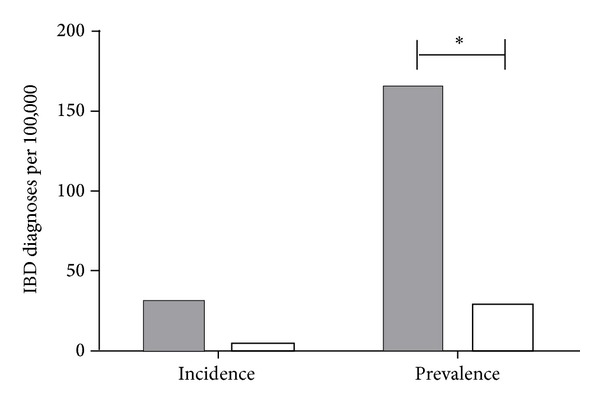
The incidence and prevalence of IBD in the pediatric population were calculated for the Sydney Children's Hospital, Randwick, catchment area for the year 2006. The prevalence of IBD amongst children with Middle Eastern ethnicity was significantly higher (*RR 5.76; CI 2.30–14.43) than prevalence amongst children with non-Middle Eastern ethnicity.

The incidence of IBD in the SCHR catchment area in 2006 for the Middle Eastern pediatrics population (aged 0–16 years) was higher (33.1 per 100 000 children per year) compared to the control group (4.3 per 100 000 children per year). The relative risk analysis, although indicating a high risk of IBD with Middle Eastern ethnicity, does not reach significance (RR 7.63, 95% CI 0.95–65.01) (Figure 2). However the prevalence of IBD in the Middle Eastern pediatric population was significantly higher at 165.4 per 100 000 children compared to the control prevalence rates of 28.7 per 100 000 children (RR 5.76, 95% CI 2.30–14.43) (Figure 2).

### 3.3. Therapy

Overall, there were no differences in the use of standard medical therapies between the groups (*P* > 0.05 for all). In addition, there was no difference in surgical management between the two groups (*P* > 0.05). However, considering the children with CD separately, the use of thiopurines was significantly higher in the Middle Eastern group for the management of CD (*P* = 0.002) (Table 5). There was no difference in use of corticosteroids, aminosalicylates, biologicals, or tacrolimus for management of CD between the groups (Table 5). There was also no difference for any of the therapies between the groups for the management of UC.

## 4. Discussion

This is the first study comparing the incidence, presentation, and management of IBD in a Middle Eastern pediatric population now residing in a “Western” country. The estimated incidence rate for IBD in this population in 2006 was among the highest reported in the pediatric literature and was almost 8 times higher than that observed for the non-Middle Eastern population in the same location. There is great variation in the literature on the difference in incidence between different populations. However our findings are consistent with those of other studies that have identified higher risk of IBD in specific groups such as South Asians and Ashkenazi Jews [7, 9].

Accurate prevalence and incidence rates of pediatric IBD for many Middle Eastern countries have not been reported in the literature [4]. It has been postulated that the low incidence of IBD in developing countries is attributed to poor sanitation and hygiene and greater exposure to microorganisms during childhood [15]. The recent rise in incidence in both developed and developing countries has coincided with improvements in hygiene over the twentieth century and the move from a lifestyle of high microbial exposure to low microbial exposure [6, 16]. Australia is a relatively young country with high levels of migration. Previous studies have reported people who emigrate to Western industrialised countries are at higher risk of developing IBD [17]. Similar findings of increased incidence upon migration have been reported in patients of South Asian origin upon emigration to Canada and the UK [9]. Interestingly, 83.3% of the Middle Eastern patients in the current study were born in Australia, adding support to the theory that the 2nd generation of immigrants to industrialised countries is most at risk of developing IBD.

We have presented point incidence and point prevalence; however, there are a number of limitations that must be considered when assessing this data. The cohort was limited to the geographical catchment area of one pediatric centre in Sydney, Australia. Taking into consideration the limitations of this dataset, the small sample size and potential confounders due to immigration, emigration, and referrals outside of the catchment area, the results presented here may have either overestimated or underestimated the incidence of IBD in this population. Ahuja and Tandon [2] suggested studies that relying on the reporting of pediatric hospitals, such as the current study, may lead to an underestimation of incidence and an overestimation of disease severity. The control cohort were matched based on age, gender, and type of disease; therefore, 24 controls were included from 406 non-Middle Eastern patients listed in the IBD database. Several patients listed in the IBD database were excluded from the incidence and prevalence calculations as they came from outside the SCHR catchment area. Although the numbers are likely to be small, it is also possible that several pediatric IBD patients, both Middle Eastern and non-Middle Eastern, from within the catchment area were attending other hospitals or were being treated as private patients and as such were not included in the SCHR database. Therefore, we propose that these initial findings indicate that a further population based cohort study is warranted.

The gender preponderance (higher number of boys) observed among these Middle Eastern children contrasts with studies of the adult IBD population where there are a slightly greater proportion of females with CD [3], although a recent report by El Mouzan et al. [18] of childhood-onset IBD in Saudi Arabia also reports a higher predominance of males with CD at 56%. Nevertheless this pattern of disease distribution (58.3% CD, 29.2% UC, and 12.5% IBD-U) is consistent with recent British, Canadian, and American studies of pediatric CD populations [19–21].

Middle Eastern children with CD had significantly less disease restricted to the colon (L2) than the controls while there were similar levels of terminal ileal disease, ileocolonic disease, and upper gastrointestinal involvement between the groups. In contrast to this, studies in other ethnic populations have found more extensive colonic disease than the general IBD population [9]. However, this is consistent with a low incidence of colonic disease and high incidence of ileocolonic disease that has been observed in a series of Kuwaiti children with IBD [22]. In the current study, excluding upper gastrointestinal involvement (L4), ileocolonic disease (L3) was the most common presentation site affected in Middle Eastern CD patients. Studies estimate that upper gastrointestinal involvement occurs in the range of 30–80% in children and less than 10% of adults with CD [23–26]. In concordance with this, a large proportion of both groups in the current study had upper GI (L4) involvement.

The comparatively high use of thiopurines in the treatment of CD in the Middle Eastern patients is suggestive of a more severe disease requiring immunosuppressive treatment. The efficacy of thiopurines in maintaining clinical remission in CD is well established with trial data supporting the introduction of thiopurines in children with moderately severe disease at diagnosis [27]. Interestingly, in the current study, the severity of disease was not reflected in the duration of symptoms, symptoms at presentation, or surgical management, as there were no differences between the two groups. However the Middle Eastern patients had higher CD activity scores and ESR at diagnosis than the controls. This finding, along with the higher thiopurine use, is suggestive of a more active disease in Middle Eastern children.

The mean age at diagnosis for this cohort of patients was 9.8 years, which was slightly lower than reported in the literature [20]. Family history appears to be one of the most important factors that confer risk for the development of IBD. No difference was established in family history rates between the Middle Eastern (22.7%) and non-Middle Eastern patients (9.1%). Recent publications of pediatric IBD in Saudi Arabia report family history rates of 15.3% [18] and 9.4% [28]. Further, the incidence of a positive family history in the Middle Eastern patients was comparable to that observed in a series of Kuwaiti children [22]. Despite this, there is variability in the family history rates reported which likely reflects the small numbers of patients in these reports. Therefore further investigation is required to determine if Middle Eastern children are at the same or greater risk of IBD than non-Middle Eastern children. Consanguinity data was not available for either the Middle Eastern or non-Middle Eastern cohorts and could not be considered when assessing family history rates.

Altered linear growth is commonly present in children at the time of presentation with IBD [29, 30]. A pediatric study in Kuwait found that growth failure was a significant problem in their patients at presentation [22]. In contrast to this, only 8.3% of the Middle Eastern children and 4.2% of controls had height for age *z*-scores indicative of stunted growth. This inconsistency may be due to the small sample size or may represent shorter symptom duration prior to diagnosis and therefore less impact upon linear growth. Active IBD may also impact adversely upon pubertal development, especially in boys [31]. Although the pubertal status of the children at diagnosis in the current retrospective study was not available, many of the children were of a prepubertal age. In addition to the limitations provided by a retrospective study design, the sample size of the current study also limited more complete full data interpretation. This was especially evident in analysis of the UC subgroup. The sample size also likely influenced the interpretation of the incidence rates between the two groups: this lack of significance may reflect a type 2 error.

In conclusion, the present study indicated that Middle Eastern patients were less likely to have disease restricted to the colon than the control children. Further the Middle Eastern children had higher CD activity at diagnosis and also required a higher incidence of immunosuppressive treatment. This data is consistent with a more severe phenotype of CD in Middle Eastern children. Although there were limitations with the dataset used to calculate point incidence and prevalence, the calculated values indicated that there is a higher point incidence and prevalence of IBD in Middle Eastern children attending the SCHR. Likely these patterns of disease in an ethnic group now resident in Australia reflect the interactions between environmental and genetic factors. Further epidemiological and genetic investigations of such populations with high incidence of disease are required to better understand the aetiology of pediatric IBD.

## Figures and Tables

**Table 1:** Group characteristics.

	Middle Eastern	Control	*P* value
Males	15 (62.5%)	15 (62.5%)	1.00
UC	7 (29.2%)	7 (29.2%)	1.00
CD	14 (58.3%)	14 (58.3%)	1.00
IBD-U	3 (12.5%)	3 (12.5%)	1.00
Age at diagnosis^#^	9.8 (4.5)	9.9 (4.4)	0.98

Data presented as number of patients (%) and compared by Fisher's exact test except ^#^where data is presented as mean (SD) and compared by unpaired *t*-test.

**Table 2:** Symptoms at presentation.

	Middle Eastern		Control		*P* value
	Yes	No	Yes	No
Abdominal pain	13	8	13	10	0.77
Diarrhoea	16	5	18	5	1.00
Mucus or blood in stools	10	11	13	10	0.76
Weight loss	10	11	8	15	0.54
Per rectal bleeding	2	19	7	16	0.14
Loss of appetite	3	18	2	21	0.66

Data compared by Fisher's exact test. Presenting symptoms not available for 3 Middle Eastern patients and 1 control patient.

**Table 3:** Blood results at diagnosis.

	Middle Eastern	Control	*P* value
^∧^ESR	31 (3–74)	18.5 (2–69)	0.02*
^∧^CRP	5 (1–198)	6 (1–62)	0.34
Platelets	392 (96)	434 (130)	0.24
Haematocrit	0.35 (0.05)	0.35 (0.05)	0.53
Albumin	35.6 (7.1)	38.6 (8.8)	0.23
^∧^ALT	12 (6–35)	18.5 (8–147)	0.03*
^∧^AST	14 (5–28)	24 (6–128)	0.01*

Data presented as mean (SD) and compared by unpaired *t*-test except  ^∧^where data is presented as median (range) and compared by Mann Whitney test. *indicates statistical significance.

**Table 4:** Disease location CD.

	Middle Eastern	Control	*P* value
L1	3	0	0.22
L2	3	11	0.01*
L3	8	3	0.12
L4	13	10	0.33

Data compared by Fisher's exact test. *indicates statistical significance.

**Table 5:** Clinical management of CD.

	Middle Eastern		Control		*P* value
	Yes	No	Yes	No
Corticosteroids	13	1	9	5	0.16
EEN	9	5	3	11	0.054
Aminosalicylates	10	4	13	1	0.33
Thiopurines	14	0	6	8	0.002*
Methotrexate	5	9	1	13	0.16
Biologicals	1	13	1	13	1.00
Tacrolimus	0	14	1	13	1.00
Surgery	4	10	2	12	0.65

Data compared by Fisher's exact test. *indicates statistical significance.
